# Amputation of Thigh for Disease of the Knee Joint

**Published:** 1860-02

**Authors:** 


2. Amputation of the Thigh for disease of the Knee Joint.—Transfusion of Blood.—John Irwin, a patient affected with caries of the knee joint, and extensive suppuration for several months for caries. This patient had been advised and urged strongly to submit to amputation, but declined. When however, it became evident that he was sinking under the disease, he consented, but delayed the operation for ten days, in which to be able to communicate with his friends. When at length, he was prepared for the operation he seemed little likely to survive it. In order to obviate the danger from loss of blood, I had an assistant hold a bowl under the member at the moment of the incision, in order to catch the blood and stir it so as to separate the fibrin. This bowl was placed in another containing water at the tempature of 98°. When the bone had been sawed, and the small vessels secured, the patient appeared to be dying from syncope. I then fixed the the tube of the transfusion syringe into the femoral artery where it had been divided upon the stump, and the syringe at the proper tempature, I charged it with the difibrinated blood which had been actively agitated so as to be of a bright red color, and threw gently into the artery, nearly all the blood which had been secured. A light tremulous movement followed and for about a minute no change was perceived, but at the end of this time the respiration and the action of the heart became more regular, and he was soon as well as before the operation. The blood thrown into the artery was just two ounces.

The points of importance in this operation I conceive to be *having the blood perfectly difibrinated*, *kept at the tempature of from 95 to 98 degrees*, *and thrown in gradually without*

*admixture of bubbles of air*. The use of difibrinated blood instead of fresh blood thrown from the vein of another person, is made in conformity with the recommendation of Dr. E. Brown Sequard, founded upon numerous experiments on animals.

The choice of the artery instead of a vein for the transfusion, was made for these reasons:—

1. It obviates the danger of fibrinous clots passing to the heart.

2. It diminishes the danger of air bubbles passing to the heart. These are the great dangers of transfusion.

This plan occurred to me as early as 1840, while attending the physiological lectures Magendi, at the college of France. I have often seen him insert the point of a syringe into an artery and when the pressure of the blood had raised the piston, push it down again so as to return the blood into vessels from the syringe without producing thereby any injurious effect upon the animal. Several times since I have seen patients on the point of expiring after amputation and other operations, in which great arteries had been divided, and the advantage of using the blood lost, and the arteries divided for the purposes of transfusion, has each time suggested itself to my mind.—But this was the first case in which the necessity of resorting to it has been so far anticipated as to induce the preparation necessary for carrying the plan into execution. This patient reacted from the effect of the operation, and for one week there seemed a prospect of recovery, but his exhausted condition and the abundant suppuration which took place from the stump, were unfavorable, and he died Jan. 20th, thirteen days after the operation.

